# Effect of a Semi-Purified Oligosaccharide-Enriched Fraction from Caprine Milk on Barrier Integrity and Mucin Production of Co-Culture Models of the Small and Large Intestinal Epithelium

**DOI:** 10.3390/nu8050267

**Published:** 2016-05-06

**Authors:** Alicia M. Barnett, Nicole C. Roy, Warren C. McNabb, Adrian L. Cookson

**Affiliations:** 1 Food Nutrition & Health Team, Food & Bio-based Products Group, AgResearch Grasslands Research Centre, Tennent Drive, Private Bag 11008, Palmerston North 4442, New Zealand; nicole.roy@agresearch.co.nz; 2 Riddet Institute, Massey University, Private Bag 11222, Palmerston North 4442, New Zealand; adrian.cookson@agresearch.co.nz; 3 AgResearch Grasslands, Palmerston North 4442, New Zealand; warren.mcnabb@agreserach.co.nz; 4 Food Assurance & Meat Quality Team, Food & Bio-based Products Group, Hopkirk Institute, Palmerston North 4442, New Zealand

**Keywords:** caprine milk oligosaccharides, Caco-2:HT29-MTX co-cultures, intestinal barrier function, mucin

## Abstract

Caprine milk contains the highest amount of oligosaccharides among domestic animals, which are structurally similar to human milk oligosaccharides (HMOs). This suggests caprine milk oligosaccharides may offer similar protective and developmental effects to that of HMOs. However, to date, studies using oligosaccharides from caprine milk have been limited. Thus, this study aimed to examine the impact of a caprine milk oligosaccharide-enriched fraction (CMOF) on barrier function of epithelial cell co-cultures of absorptive enterocytes (Caco-2 cells) and mucus-secreting goblet cells (HT29-MTX cells), that more closely simulate the cell proportions found in the small (90:10) and large intestine (75:25). Treatment of epithelial co-cultures with 0.4, 1.0, 2.0 and 4.0 mg/mL of CMOF was shown to have no effect on metabolic activity but did enhance cell epithelial barrier integrity as measured by trans-epithelial electrical resistance (TEER), in a dose-dependent manner. The CMOF at the maximum concentration tested (4.0 mg/mL) enhanced TEER, mucin gene expression and mucin protein abundance of epithelial co-cultures, all of which are essential components of intestinal barrier function.

## 1. Introduction

Human milk oligosaccharides (HMOs) have many beneficial functions, including the selective enrichment of the intestinal microbiota with beneficial bifidobacteria [1]; anti-infective properties due to their structural similarity to host cell surface receptors and mucin proteins [2]; promoting the immune system; modulating the normal growth, differentiation, and apoptosis of intestinal epithelial cells [3,4]; and enhancing intestinal epithelial barrier function [1]. Although some HMOs can be chemically synthesised, an alternative source of natural lactose-derived oligosaccharides is from ruminant milk. The composition of ruminant milk varies between different species and breeds, and also throughout lactation [5]. Caprine milk contains substantially less oligosaccharides than human milk (3–20 g/L) [6,7] at 0.25–0.30 g/L but has 5–8 times more than bovine milk (0.03–0.06 g/L) and 10 times more than ovine milk (0.02–0.04 g/L) [2]. Although milk from these ruminant species contains a variety of neutral and acidic oligosaccharide structures, the overall oligosaccharide profile of caprine milk is more similar to human milk than either bovine or ovine milk [2]. 

To date only a limited number of studies have investigated the effects of caprine milk oligosaccharides on intestinal barrier function. The intestinal barrier has two components: (1) the intrinsic barrier that consists of the continuous single layer of intestinal epithelial cells; and (2) the extrinsic mucus barrier which is a combination of secretions such as mucins, and trefoil peptides from goblet cells within the epithelial layer [8,9,10]. Four main epithelial cell types; Paneth cells, enteroendocrine, absorptive enterocytes, and goblet cells; make up the epithelium [11,12]. Absorptive cells are the most abundant epithelial cell type in the intestinal epithelium, while the proportion of goblet cells, among other epithelial cell types, increases from the duodenum (4%) to the distal colon (16%–24%) [11,12,13,14]. Enteroendocrine cells represent less than 1% of epithelial cells [15]. An important component of the intrinsic barrier is the intercellular junctional complexes [16] that regulate the entry of luminal nutrients, ions and water while restricting pathogen entry and thus regulate the barrier function of the epithelium [17]. The intestinal mucus barrier forms a bilayer; a loose outer layer that provides an environment for the commensal microbiota and the inner adherent layer that is devoid of them [8,18,19]. In addition, membrane-bound mucins at the apical surface of the epithelium are a dominant glycoconjugate component of the glycocalyx, which forms an intermediate layer of defence between the extrinsic and intrinsic barriers [8]. The hydrophilic nature of the mucus and glycocalyx provides a physical barrier that protects the epithelial cells by acting as a diffusion barrier [20] between the luminal contents and the epithelial lining [21]. An abnormal mucus layer may lead to both acute and chronic intestinal diseases and has been shown to be associated with colitis in a murine model [22,23]. Additionally, a dysfunctional epithelial barrier is a critical factor in predisposition to intestinal diseases such as: food allergy, inflammatory bowel diseases, and celiac disease [24] and is also associated with autoimmune diseases in other parts of the body [25,26].

A recent study has shown that oligosaccharides from caprine milk normalise the expression levels of selected mucin genes in a dextran-sodium-sulfate-induced rat model of colitis [27]. In contrast, another study determined oligosaccharides from caprine milk modulate the expression of selected mucin genes and trefoil factors in HT29-MTX mono-cultures [28]. However, these studies did not investigate the effects of these milk constituents on mucin protein abundance and thus the relationship between mucin gene expression and mucin protein abundance was not characterised. The underlying mechanisms of action of oligosaccharides on mucin gene/protein expression are not fully understood, although it is known that oligosaccharides interact with glycan binding proteins differentially expressed by epithelial cells [29], and that neutral oligosaccharides, but not acidic oligosaccharides, can be found in the intracellular compartment of Caco-2 cells [30]. Other *in vitro* studies have shown the effects of oligosaccharides on cellular proliferation [4], transport mechanisms [30], and mucin gene expression [28]. However, the cellular models used were representative of one cell type and thus may not be representative of the impact oligosaccharides have on the barrier integrity and the surface mucus layer of the intestinal epithelium *in vivo*.

In this study we hypothesised that a caprine milk oligosaccharide-enriched fraction (CMOF) would enhance epithelial barrier integrity, mucin mRNA levels and mucin protein abundance of the small and large intestinal epithelium to improve the intrinsic and extrinsic intestinal barrier function. We used intestinal epithelial cell mono- and co-culture models which incorporated both absorptive enterocytes (Caco-2) and mucin secreting goblet cells (HT29-MTX), the two most abundant cell types found in the intestinal epithelium, in ratios that represented the small (90:10 Caco-2:HT29-MTX) [13] and large intestine (75:25 Caco-2:HT29-MTX), to characterise the impact of CMOF on cell metabolic activity and epithelial barrier integrity (as measured by trans-epithelial electrical resistance (TEER)). The effects of the CMOF on the levels of mucin gene expression and mucin protein abundance were also measured. MUC4, a membrane-bound mucin and MUC2, a secreted mucin were selected as they are the major mucins expressed in the human small and large intestine [31,32] while MUC5AC (another secreted mucin), although preferentially expressed in the stomach [33] and respiratory tract [31], is the major mucin expressed by HT29-MTX cells [9].

## 2. Experimental Section

### 2.1. Composition of Caprine Milk Oligosaccharide-Enriched Fraction

The CMOF was kindly provided by Caroline Thum (AgResearch, Grasslands, Palmerston North, New Zealand) [34]. The oligosaccharide-enriched fraction was isolated as described previously [34]. The carbohydrate composition of the CMOF after purification (Table 1) and the oligosaccharide profile of the CMOF (Table 2) were determined by high-performance liquid chromatography (HPLC) analysis and HPLC-mass spectrometry as described by Thum *et al.* [34]. 

### 2.2. Stock Solutions of CMOF for Intestinal Epithelial Cell Based Assays

The CMOF (50 mg/mL) for all epithelial cell culture experiments was suspended in phosphate buffered saline (PBS) pH 7.2, and filter sterilised (0.22 µm filters; Millipore Australia Pty Ltd., Sydney, Australia). For all cell based assays the stock CMOF solution was diluted with non-supplemented tissue culture medium (Dulbecco’s Modified Eagles Medium (DMEM) Glutamax containing 1.0 g/L glucose and 0.8 g/L l-alanyl-l-glutamine; Life Technologies, Penrose, Auckland, New Zealand) until the desired concentration was obtained for the assay. 

### 2.3. Cell Lines

The human colorectal adenocarcinoma cell line Caco-2 (HTB-37) was obtained from the American Type Culture Collection (ATCC, Manassas, VA, USA) at passage 18 and used in experiments at passage 28 to 33. It was critical to use cells within defined passage numbers, as phenotypes of Caco-2 cells can change over time [35]. The human colon adenocarcinoma cell line HT29 (HTB-38; ATCC) previously adapted with 10^−7^ *M* methotrexate (MTX) was kindly provided by Rachel Anderson (AgResearch, Grasslands) at passage 9. These HT29-MTX cells were further adapted with 10^−6^ *M* MTX as described previously [36,37] and used in experiments from passage 18 to 31. 

### 2.4. Cell Culture

Caco-2 and HT29-MTX cells were grown separately in tissue culture flasks (Corning, Lindfield, Sydney, Australia) in complete medium consisting of DMEM Glutamax supplemented with 10% (*v*/*v*) foetal bovine serum (FBS; Life Technologies) and 1% (*v*/*v*) Penicillin-Streptomycin (Pen-Strep; 10,000 units/mL Penicillin and 10 mg/mL Streptomycin; Sigma-Aldrich, Auckland, New Zealand).

Cells were sub-cultured weekly when they reached 80% confluence, detached with TrypLE Express (Life Technologies, Auckland, New Zealand) and seeded at a 1:5 dilution into a new 75 cm^2^ flasks (Corning). The culture medium was initially replaced after 24 h with subsequent medium changes every 48 h. The cells were maintained at 37 °C in a 5% CO_2_, 95% air/water saturated atmosphere. For experimental studies, Caco-2 and HT29-MTX cells were stained with trypan blue, counted using the Countess automated cell counter (Life Technologies), resuspended at ratios of 100:0 (Caco-2:HT29-MTX), 90:10 (to simulate the small intestine [13]), 75:25 (to simulate the large intestine [13]) and 0:100 and seeded at a density of 6.3 × 10^4^ cells per cm^2^ in all culture vessels used. 

### 2.5. Measurement of Metabolic Activity of Intestinal Epithelial Cell Cultures

The metabolic activity of Caco-2:HT29-MTX cells (100:0, 90:10, 75:25, and 0:100) was determined by the 4-[3-(4-Iodophenyl)-2-(4-nitrophenyl)-2H-5-tetrazolio]-1,3 benzene disulfonate (Wst-1) colourimetric assay (Roche, Auckland, New Zealand). Metabolic activity, which directly correlates to cell proliferation, was quantified by absorbance at 450 nm with a reference wavelength of 650 nm (FlexStation 3 Benchtop Multi-Mode Microplate Reader; Molecular Devices, Sunnyvale, CA, USA).

In 96-well tissue-culture plates (Corning, Lindfield, Sydney, Australia), pre-confluent (48 h post-seeding) and post-confluent (21 days post-seeding) Caco-2:HT29-MTX cells (100:0, 90:10, 75:25, and 0:100) were incubated for 24 h in non-supplemented media (DMEM Glutamax only; no FBS or Pen-Strep) or medium containing CMOF at, 0.4, 1.0, 2.0, and 4.0 mg/mL. At these concentrations the oligosaccharide component of the CMOF was 0.1, 0.25, 0.5, and 1.0 mg/mL respectively. Each concentration of CMOF and control (non-supplemented) was tested in 10 replicates. All cells were cultured in the presence of test solutions for 24 h at 37 °C in an atmosphere of 5% CO_2_ in air. In the last hour of incubation the medium was replaced with medium containing Wst-1 reagent (1:10 medium: Wst-1 reagent). Additional wells in each plate containing medium and Wst-1 reagent only (without cells) were processed in parallel and used as reference blanks [38,39]. For each cell culture the assay was repeated on three separate occasions. Cell metabolic activity was expressed as a percentage of the control.

### 2.6. Trans-Epithelial Electrical Resistance Assay

24 h prior to TEER measurements, mucin gene, and mucin protein assays, the culture medium was gently removed and replenished with non-supplemented (no FBS or Pen-Strep) culture medium in each well with the liquid flowing slowly over the surface of the monolayers. The culture medium was replaced with serum- and antibiotic-free medium to eliminate any interference from extraneous proteins or hormones [40]. After 10 min the cell culture medium was removed (wash 1) and replenished with fresh medium. After an additional three gentle washes (at hourly intervals) warm cell culture medium was replenished and monolayers incubated for 24 h.

Caco-2:HT29-MTX cultures (100:0, 90:10, 75:25, and 0:100) were seeded onto 12 mm diameter, 0.4 μm^2^ pore size, PET Transwell inserts (Corning) and cultured as described above.

Post-confluent monolayers were prepared 24 h before (day 20 post-seeding) the TEER assay as described above. After 24 h incubation, initial resistance readings were obtained (EndOhm Culture cup connected to an EVOM voltohmmeter (World Precision Instruments, Sarasota, FL, USA)) for all monolayers, after which point the medium was replaced with non-supplemented medium in the basal compartment and in the apical compartment either non-supplemented medium (control), or CMOF-supplemented medium at increasing concentrations (0.4, 1.0, 2.0, and 4.0 mg/mL) was added. The resistance across each cell monolayer was measured and the percentage change in TEER compared to initial TEER for each insert was calculated as described previously [41]. Experiments were undertaken in triplicate (three successive passages of cells), each with three replicates per treatment.

### 2.7. Quantification of Mucin Gene Expression of Intestinal Epithelial Cell Cultures

Caco-2:HT29-MTX cultures (100:0, 90:10, 75:25, and 0:100) were seeded into 12-well cell culture plates (Corning) in complete culture medium. Twenty days post-seeding monolayers were prepared as described above. After 24 h the spent medium (SM) was removed and monolayers washed gently four times with PBS. Pre-warmed, PBS-supplemented (control) standard medium or medium supplemented with 4.0 mg/mL CMOF was gently added to the monolayers and cultures incubated for 12 h. 

The expression of mucin genes in Caco-2:HT29-MTX cultures (100:0, 90:10, 75:25, and 0:100) treated with 4.0 mg/mL CMOF for 12 h was quantified using TaqMan real-time PCR. All reagents were obtained from Applied Biosystems (Foster City, CA, USA) unless otherwise stated. The expression of these genes in reference samples (untreated controls) was also quantified. The genes quantified were; *MUC2*, *MUC4*, and *MUC5AC*.

After 12 h incubation the SM was removed and monolayers were lysed with 1 mL of Tri-reagent (Invitrogen, Auckland, New Zealand). The total RNA from each cell lysate was isolated using the RiboPure RNA isolation kit as per the manufacturer’s protocol, with the following modifications; 1-bromo-3-chloropropane, 100 µL, (Sigma-Aldrich) was used in place of chloroform, two wash steps were completed and the total RNA was eluted in two volumes of 30 µL. RNA integrity was measured using an Agilent 2100 Bioanalyser (Agilent Technologies, Santa Clara, CA, USA). 

For real-time PCR analysis, 1.5 μg of total RNA was reverse transcribed into cDNA using a high-capacity RNA-to-cDNA Kit (Applied Biosystems) according to the manufacturer’s instructions. Expression levels of three mucin genes (*MUC2*, *MUC4*, and *MUC5AC*; TaqMan assay IDs Hs.PT.56a.26485553, Hs.PT.56a.5039491 and Hs.PT.56a.25473826, respectively), relative to the reference gene hypoxanthine phosphoribosyltransferase [42,43] (*HPRT1*; Hs.PT.39a.22214821, NM_000194.1), were determined using TaqMan probes on the Rotor-Gene 6000 real-time thermal cycler (Corbett Life Science, Concord, Australia). All PCRs (no template controls, untreated, and treated samples) were prepared as triplicate 10 μL reactions as described previously [44]. The thermal profile used was 95 °C for 180 s followed by 40 cycles 95 °C for 3 s and 60 °C for 30 s. The data were normalised to the reference gene and analysed for expression level changes using Relative Expression Software Tool (REST) 2009 software (version 2.0.13; Qiagen, Valencia, CA, USA). Experiments in triplicate were completed (three successive passages of cells), each with three replicates per treatment. Each sample was analysed in triplicate by qPCR.

### 2.8. Mucin Protein Abundance

The total production of mucin proteins consists of mucins in both the cell lysate (CL) and SM. Therefore, in the present study, the total production of MUC2, MUC4 and MUC5AC mucin protein abundances was calculated as the sum of the mucin protein in CL and SM and measured by indirect enzyme linked immunosorbent assay (indirect ELISA). Post-confluent cell cultures were prepared and treated with 4.0 mg/mL CMOF as described above. At the end of the designated incubation period (12 h post-wash) SM samples were collected and proteinase inhibitors (10x concentrate in PBS; Complete mini (EDTA-free); Roche, Indianapolis, IN, USA) added to the samples. Cells were lysed with lysis buffer (1% Triton X-100; Sigma-Aldrich and proteinase inhibitors 1x concentration in PBS) for 10 min. All samples were centrifuged at 4000× *g* for five min at 4 °C and stored at −80 °C until analysis. 

Total mucin production was calculated as the sum of mucin proteins in the CL and SM. The total protein concentration in samples was determined using the BCA protein assay kit (Thermo Fisher Scientific NZ Limited, North Shore City, New Zealand) according to the manufacturer’s instructions and using BSA as standards.

For the ELISA, 50 μL of SM sample was serially diluted with carbonate-bicarbonate buffer (pH 9.6), in medium protein-binding, flat bottom, enzyme immunoassay/radioimmunoassay (EIR/IRA) 96-well plates (*In Vitro* Technologies, Auckland, New Zealand). CL samples were diluted in carbonate-bicarbonate buffer and 50 μL (at 5 ng/μL total protein) added to EIA/RIA plates. After overnight incubation at 4 °C, samples were decanted and wells blocked with 150 μL of filter sterilised (0.22 µm filters; Millipore Australia Pty Ltd, Sydney, Australia) 3% BSA fraction V (Sigma-Aldrich) in PBS for 1 h at room temperature. The blocking agent was decanted and 50 μL of either MUC2 mouse mono-clonal antibody (clone 4A4, 1:250; Creative Biomart, New York, NY, USA), MUC4 mouse mono-clonal antibody (clone 5B12, 1:500; Abnova, Taipei, Taiwan) or MUC5AC mouse mono-clonal antibody (clone 2H7, 1: 250; Abnova) in 1% BSA was added to wells and plates incubated at room temperature for 1 h. Primary antibody was decanted and all plates were washed once with 0.1% PBS-Tween (Tween 20; Sigma-Aldrich) followed by an additional three washes with PBS. After all washing steps, 50 μL horseradish peroxidase-rabbit anti-mouse immunoglobulin G conjugate (Abcam, Cambridge, UK; 1:5000 dilution for MUC2 and MUC5AC and 1:10,000 dilution for MUC4) in 1% BSA was dispensed into each well. After 1 h, plates were washed once with 0.05% PBS-Tween and three times with PBS. Colour reaction was developed with 3,3′,5,5′-tetramethylbenzidine (TMB) peroxidase solution (100 μL/well; Invitrogen, Auckland, New Zealand) for 0.5 h and stopped with 2N H_2_SO_4_ (Reagent grade sulphuric acid; Sigma-Aldrich) (50 μL/well). Absorbance was read at 450 nm (FlexStation 3 Benchtop Multi-Mode Microplate Reader; Molecular Devices, Sunnyvale, California, USA) using the well scan option. The concentration of MUC2, MUC4 and MUC5AC in all samples was calculated from standard curves using MUC2 (Creative Biomart, New York, NY, USA), MUC4 (Abnova) and MUC5AC (Abnova) recombinant proteins as standards. There was no cross reactivity of the mucin mono-clonal antibodies to the different recombinant proteins. The abundance of mucin proteins was calculated as nanograms of mucin protein per microgram of total protein from each of the monolayers. Experiments were completed in triplicate (three successive passages of cells), each with three replicates per treatment. Each sample was analysed in duplicate by indirect ELISA. 

### 2.9. Statistical Analysis

Statistical analysis was undertaken with Sigma-Stat 12.0 software, except the real-time PCR data which was analysed using Relative Expression Software Tool 2009 (Version 2.0.13, Qiagen) with efficiency correction [45]. For the Wst-1 metabolic activity bioassay and the ELISA protein abundance assay, treatments were compared by using an analysis of variance (ANOVA). Tukey *post hoc* test was used to determine whether the total protein abundance of control mono- and co-cultures differ significantly. For the TEER assay treatments were compared using a repeated measure ANOVA. Differences were considered statistically different at values of *p* < 0.05. 

## 3. Results

### 3.1. Metabolic Activity of Epithelial Cell Mono- and Co-Cultures in Response to CMOF

**Figure 1:**
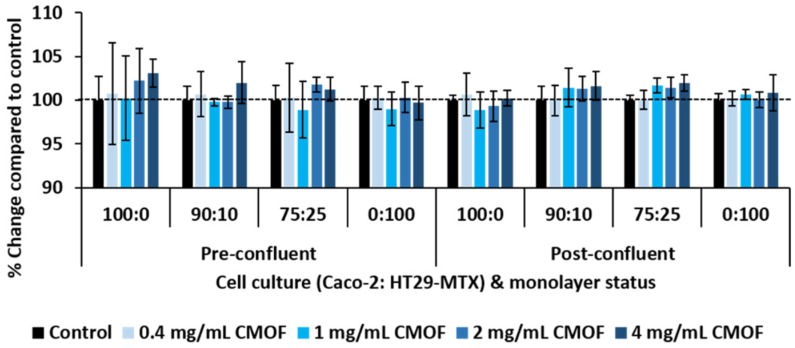
Effect of incubation of a caprine milk oligosaccharide-enriched fraction (CMOF) on the metabolic activity of mono- and co-cultures of Caco-2 and HT29-MTX cells. The metabolic activity of pre-confluent (48 h post-seeding) and post-confluent (day 21 post-seeding) Caco-2:HT29-MTX cell cultures after 24 h incubation as a percentage of control as measured after the Wst-1 assay. Values are means (±SEM) for three experiments (10 samples per treatment per experiment).

Incubation with the CMOF at all concentrations for 24 h had no significant effect on the metabolic activity, as measured by the Wst-1 assay, of pre- and post-confluent 100:0, 90:10, 75:25, and 0:100 Caco-2:HT29-MTX mono- and co-cultures when compared to control (Figure 1). 

### 3.2. Impact of CMOF on TEER of Epithelial Cell Monolayers

The average (± SEM) initial TEER values (Ω·cm^2^) of each of the cell cultures recorded prior to the introduction of the CMOF were 1070 ± 25, 272 ± 7.3, 163 ± 2.1, and 21 ± 0.3 for 100:0, 90:10, 75:25, and 0:100 (Caco-2:HT29-MTX) respectively.

**Figure 2:**
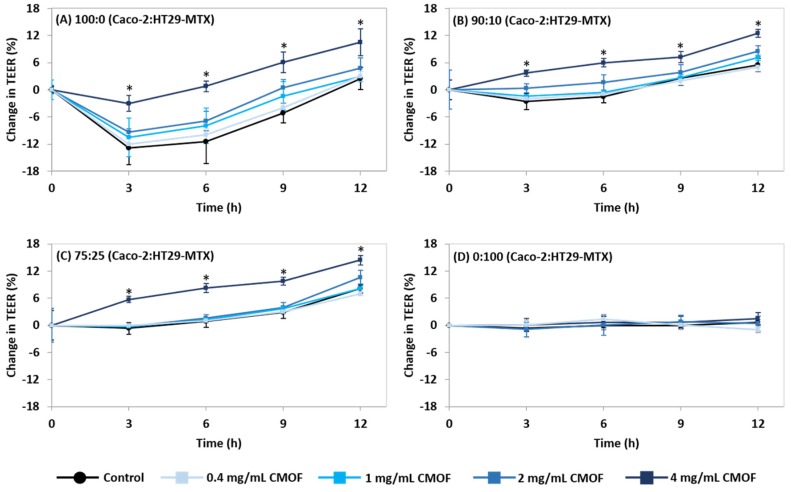
Impact of a caprine milk oligosaccharide-enriched fraction (CMOF) on trans-epithelial electrical resistance (TEER) of post-confluent monolayers over time. The change in TEER as the percentage change compared with initial TEER for (**A**) 100:0, (**B**) 90:10, (**C**) 75:25, and (**D**) 0:100 Caco-2:HT29-MTX cultures. Values are means (±SEM) for three experiments (three samples per treatment per experiment). * Significantly different to respective controls *p* < 0.05.

Incubation with 4 mg/mL CMOF significantly increased (*p* < 0.05) TEER of the 100:0, 90:10, and 75:25 Caco-2:HT29-MTX cultures in comparison to respective controls at all the time points (Figure 2). At concentrations ≤2.0 mg/mL the change in TEER was not significantly different to control monolayers at any time point. There was no change in TEER at any time point for 0:100 cultures in response to the CMOF at any concentration (Figure 2). 

The observed drop in TEER of control and CMOF-treated 100:0 mono-cultures between 0 and 3 h was likely due to the monolayers being disturbed by the change in media during the sample addition after the initial readings [46]. The 90:10 and 75:25 Caco-2:HT29-MTX co-cultures were less affected by the change in media (no significant initial drop in TEER) and had less variation between replicates (lower SEM values).

### 3.3. Mucin Gene Expression of Epithelial Cell Cultures in Response to CMOF

**Figure 3:**
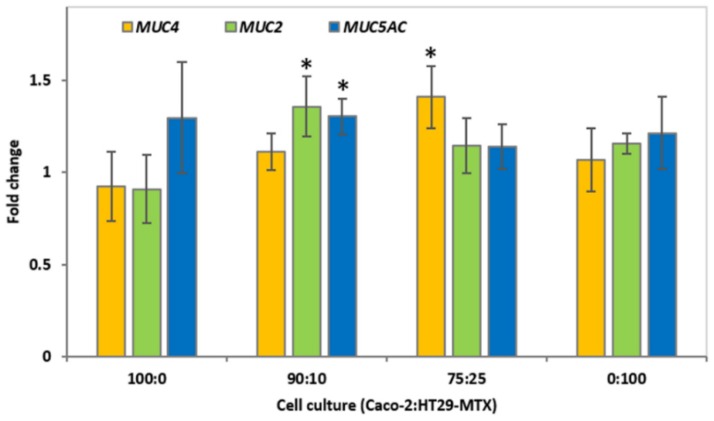
Relative fold change of MUC mRNA from epithelial cell cultures incubated with 4 mg/mL caprine milk oligosaccharide-enriched fraction (CMOF). The expression of mucin genes *MUC4*, *MUC2* and *MUC5AC* by 100:0, 90:10, 75:25, and 0:100 Caco-2:HT29-MTX cell cultures after 12 h incubation with 4.0 mg/mL CMOF, compared to respective control monolayers. The data are expressed as the mean fold change (±SEM) of three replicates across three independent experiments. A statistically significant difference in fold change is indicated by * (*p* < 0.05).

The expression of selected mucin genes was determined in epithelial cell cultures incubated with 4.0 mg/mL CMOF for 12 h (Figure 3). This concentration of CMOF was selected as it was shown to increase TEER compared to control at all the time points for cell cultures containing Caco-2 cells.

Treatment with 4.0 mg/mL CMOF resulted in a moderate increase (fold change > 1.2) in *MUC5AC* mucin gene expression for 100:0 (1.3 ± 0.3) and 0:100 (1.2 ± 0.2) Caco-2:HT29-MTX mono-cultures, although this was not significant (*p* > 0.05) (Figure 3). Large variations in gene expression may be observed when control tissue culture cells are compared to a disease model [46]. However, the response of a given dietary component on gene expression can be minimal [47]; thus, in this study a relatively low cut-off of a 1.2 fold-change was selected because the intention was to compare untreated tissue culture cells with those where a potential prebiotic was added. 

For 90:10 and 75:25 co-cultures there was a significant (*p* < 0.05), but moderate, increase in the expression of mucin genes after incubation with 4.0 mg/mL CMOF compared to respective controls. The 90:10 co-cultures had a significant increase (*p* < 0.05) in expression of *MUC2* and *MUC5AC*, while in 75:25 co-cultures *MUC4* expression was significantly increased (*p* < 0.05). 

### 3.4. Mucin Protein Abundance of Epithelial Cell Cultures in Response to CMOF

The total protein abundance of control and CMOF treated mono- and co-cultures was determined. There was a significant difference (*p* < 0.05) in the total protein abundance between control 100:0 (0.701 ± 0.013 mg) mono-cultures and control 90:10 (0.857 ± 0.017 mg) co-cultures, control 75:25 (0.892 ± 0.006 mg) co-cultures, and control 0:100 (0.950 ± 0.015 mg) mono-cultures. In addition there was a significant increase (*p* < 0.05) in total protein of control 0:100 mono-cultures when compared to control 90:10 co-cultures. In contrast, there was no significant difference (*p* > 0.05) in the total protein abundance between control and CMOF-treated cultures with the same cellular compositions (CMOF-treated 100:0 (0.743 ± 0.010 mg), CMOF-treated 90:10 and 75:25 co-cultures (0.908 ± 0.017 mg and 0.895 ± 0.012 mg respectively) and CMOF-treated 0:100 mono-cultures (0.986 ± 0.009 mg).

**Figure 4:**
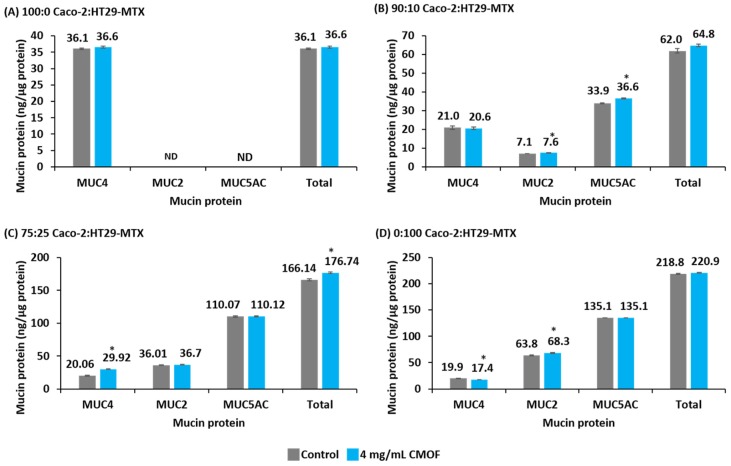
The abundance of the individual mucin proteins (MUC4, MUC2 and MUC5AC) and the total mucin protein abundance from post-confluent (21 days post-seeding) control Caco-2:HT29-MTX mono- and co-cultures (ng/µg total protein) and those incubated with 4.0 mg/mL caprine milk oligosaccharide-enriched fraction (CMOF) for 12 h. Results are expressed as the mean abundance (ng/µg of total protein) (±SEM). * *p* < 0.05 compared to respective control.

For the 100:0 Caco-2:HT29-MTX cultures incubated for 12 h with 4.0 mg/mL CMOF there was no significant increase (*p* > 0.05) in the abundance of MUC4 protein compared to control (Figure 4). In this cell culture (100:0) the secreted mucins MUC2 and MUC5AC were not detected in either the CL or SM samples. 

Compared to respective control monolayers, the total mucin protein abundance of 90:10 co-cultures was unchanged after incubation with 4.0 mg/mL CMOF for 12 h, even though there were significant increases (*p* < 0.05) in the abundance of both of the secreted mucin proteins MUC2 and MUC5AC.

For the 75:25 co-cultures, incubation with 4.0 mg/mL CMOF for 12 h resulted in a significant increase (*p* < 0.05) in the abundance of the membrane-bound mucin MUC4, which was consistent with the observed increase in *MUC4* transcription.

The total abundance of mucin protein from control 0:100 mono-cultures and those incubated with 4.0 mg/mL CMOF for 12 h were similar (*p* > 0.05). However, there was a significant decrease (*p* < 0.05) in the abundance of MUC4 mucin protein which was coupled with a significant increase (*p* < 0.05) in the abundance of MUC2 mucin protein in monolayers exposed to 4.0 mg/mL CMOF (Figure 4).

## 4. Discussion

To examine the beneficial effects of a CMOF on epithelial barrier function intestinal cell Caco-2 and HT29-MTX co-cultures having a similar composition to human small (90:10 [13]) and large (75:25 [13]) intestine *in vivo* were established. Addition of the CMOF was shown to enhance mucin gene expression and mucin protein abundance and also increase epithelial barrier integrity as measured by TEER. 

The TEER values for the Caco-2 mono-cultures obtained in this study (average of 1070 ± 25 Ω·cm^2^) are not directly comparable to those of published reports because TEER values associated with Caco-2 mono-cultures may vary as much as 20 fold (80–1420 Ω·cm^2^) [48]. Variations in TEER measurements may arise due to differing culture parameters; the passage number of the cells; the age and the stage of differentiation of the cells; the type of culture medium used; the seeding density of the cells and the type of support the cells are cultured on [49]. Despite this, previous studies have indicated that the incorporation of HT29-MTX cells into cultures of Caco-2 cells decreases TEER [13,49,50] and that Caco-2 mono-cultures are associated with TEER values which far exceed those found *ex vivo* [51,52,53,54]. However, average TEER values obtained for the 90:10 and 75:25 co-cultures (272 (±7.3) and 163 (±2.1) Ω·cm^2^ respectively) were comparable to *ex vivo* TEER values (8.6–400 Ω·cm^2^) [51,52,55,56] of human intestinal epithelium. 

In this study it was shown that the CMOF modulated TEER in a dose dependent manner when added to cell cultures containing Caco-2 cells. No effect was observed on TEER upon addition of the CMOF to HT29-MTX mono-cultures. However, the inclusion of the CMOF decreased TEER of 100:0 cultures after three hours which is similar to that reported previously for Caco-2 mono-cultures upon exposure to the purified non-digestible saccharide fructo-oligosaccharide [57]. In contrast to that of fructo-oligosaccharides, inclusion of the CMOF reduced the drop in resistance that was observed after the addition of the basal medium, a result which is more similar to that reported for a bovine colostral whey fraction [58]. In addition to increasing TEER of porcine epithelial jejunal cells, the bovine colostral whey fraction also induced an increase in occludin protein expression in piglets [58]. Occludin is an important integral membrane protein of tight junctions which are a major component for maintaining barrier function [58]. Maintenance of intestinal barrier function is important in human health because barrier dysfunction is a major factor contributing to the predisposition to intestinal inflammatory diseases [24].

The mechanism of action of the CMOF on TEER was not investigated in this study, however previous reports have indicated acidic and neutral oligosaccharides induce phosphorylation of the epidermal growth factor receptor (EGFR) in HT29 and Caco-2 cells resulting in the activation of the Ras/Raf/MEK1/ERK1 (Rat sarcoma/rapidly accelerated fibrosarcoma/Mitogen-activated protein kinase, extracellular regulated kinase kinase/Extracellular regulated kinase) pathway [59]. In Caco-2 cells the C-terminal domain of occludin binds directly to ERK1 suggesting that this direct interaction may play a crucial role in the regulation of the tight junction protein complex [60]. In addition, the increase in TEER of Madin Darby canine kidney cell monolayers was associated with activation of Raf-1/ERK1/2 and the expression and localisation of claudin-4, a component of tight junction strands and an important regulator of tight junction permeability [61]. 

In this study incubation with 4.0 mg/mL CMOF differentially modulated mucin gene expression levels and mucin protein abundance in cell cultures, however, changes in mucin gene expression and protein production were not simply a consequence of increased metabolism as indicated by results from the Wst-1 assay.

Unlike each respective mono-culture, 90:10 and 75:25 Caco-2:HT29-MTX co-cultures exhibited moderate increases in the transcript levels of mucin genes after 12 h incubation with the CMOF. Any potential interactions between the contrasting cell types (enterocyte and goblet cell) during co-culture remain to be established. It has been observed previously that co-culture systems, although not completely replicating the cellular microenvironment, enable the study of multicell interactions, such as the effects of soluble factor signalling and produce a more measureable response in the induction of gene expression [62]. 

Although there were only moderate changes in mucin transcript levels previous studies have noted that nutritional-induced changes in gene expression are less than the described effects observed in various diseases [63], and the lesser changes are an integral part of the physiological response [64]. These subtle changes may occur through the interactions of multiple genes, via signalling pathways or other functional relationships and that modulation in similarly behaving genes with similar biological functions can be of functional significance [64]. This is particularly relevant when considering that for the 90:10 co-culture there was an increase in the transcript levels of *MUC2* and *MUC5AC* both of which are encoded within the same cluster at chromosome location 11p15.5 [65]. 

For the 90:10 and 75:25 co-cultures, similar expression patterns were observed between mucin mRNA transcripts and mucin protein abundance after incubation with the CMOF. A relationship between mucin protein abundance and gene expression has been observed previously and may be related to the processes involved in mucin secretion [66,67]. For example, unregulated, constitutive secretion of mucins is dependent upon the continuous movement of mucin granules from the golgi to the apex of the cell and is coupled with the corresponding increase in *MUC* gene expression to replenish the intracellular mucin pool [40,68]. A similar action can be found in other secretory cells such as pancreatic β cells, which respond to changes in blood glucose by first secreting insulin and next increasing insulin biosynthesis [40].

In this study the secreted mucin proteins MUC2 and MUC5AC were not detected in samples from the 100:0 mono-cultures. In contrast, Wan *et al.* [69] detected the MUC5AC mucin protein in 100:0 mono-culture samples. Why this mucin protein was not detected in this study may be related to the antibody used. In this study the MUC5AC antibody used was raised against a partial recombinant MUC5AC mucin protein and recognises an amino acid sequence in the C-terminal domain which is downstream of the GPDH cleavage site. By comparison Wan *et al.* [69] used a MUC5AC antibody that was raised against the peptide core of mucin isolated from the fluid of a human ovarian cyst and recognises the M1-a epitope of gastric mucins [70]. Although the antibodies used in the two studies recognise different regions of the MUC5AC mucin protein, the rationale for the antibody used in this study was the availability of a recombinant protein that could be used to quantify the abundance of this protein from the cell samples. The mono-clonal antibody used in this study may not detect MUC5AC protein from Caco-2 cells because: (1) of an absence of MUC5AC in the Caco-2 cell population; (2) MUC5AC was only present at levels below the sensitivity of the ELISA; or (3) the MUC5AC mucin epitope of Caco-2 cells was not recognised by this mono-clonal antibody. Consequently, even though there was a moderate increase in the expression of *MUC5AC* in the CMOF-treated 100:0 mono-cultures, a resultant change in protein level could not be determined.

In the 90:10 co-culture, there was a much greater reduction in MUC4 protein abundance when compared to the 100:0 mono-culture than could be attributed to the change in cell composition. Whether this observed decrease was as a result of an interaction between the cell types remains to be determined. However, it could be hypothesised that signalling molecules produced by one cell type may have a regulatory impact on protein production of the other cell type. In CMOF-treated 90:10 co-cultures there was an increase in the abundance of both the secreted mucins (MUC2 and MUC5AC) although the overall total abundance of mucin protein was unchanged. In the 75:25 co-culture, the abundance of the membrane-bound mucin MUC4 was increased by approximately 50% compared to the control sample. These results may suggest subtle changes to the composition of either the mucus layer itself or the glycoproteins of the glycocalyx. Whether these changes influence the physiochemical or barrier properties of the mucus barrier requires further attention. It is possible that changes in mucin secretion may affect interactions with the extracellular environment, which could directly or indirectly influence interactions with other membrane components [71].

The mucus gel layer formed by secreted mucins maintains the integrity of the gastrointestinal mucosa surface and acts as a medium for protection, lubrication, and transport between luminal contents and the epithelial lining [21]. Membrane-bound mucins, which are anchored to the apical surface, are a major component of the glycocalyx and present a glycoarray in the extracellular space, which is available for a wide range of interactions in the luminal environment [72]. In addition to providing a physical barrier, membrane-bound mucins are also involved in intracellular signalling events [8]. However, increased expression of membrane-bound mucins, such as MUC4, can be associated with a loss of interaction between neighbouring cells, resulting from a re-localisation of E-cadherin from adherens junctions at the lateral membrane of the cells to the apical membrane [73]. There was an increase in MUC4 abundance after incubation with the CMOF in the 75:25 co-cultures, however TEER was also increased compared to control, which suggests that, even though MUC4 abundance was increased, there were no detrimental effects on tight junction integrity between cells.

The molecular mechanism by which the CMOF modulates mucin dynamics was not investigated in this study, however it could be hypothesised that activation of EGFR by oligosaccharides in the CMOF may result in the initiation of the mitogen-activated protein kinase (MAPK) signalling cascade and mucin production similar to that observed for neutral and acidic oligosaccharides [74,75]. In HT29-Cl.16E cells activation of the Ras/Raf/MEK1/ERK1 signalling cascade stimulates mucin secretion causing the release of a stored pool of mucins, although this pathway acts on the secretory process itself without interfering with the transcription of mucin genes [76]. In the colon adenocarcinoma LS174T cell line, galacto-oligosaccharides (8.0 mg/mL) stimulates the increased expression of MUC2 but not as a consequence of intracellular signalling through an NFĸB-mediated inflammatory cascade but rather as a consequence of direct interaction with goblet cells through an unknown mechanism [21].

At the maximum concentration of the CMOF used in this study (4.0 mg/mL) the metabolic activity and thus proliferation of pre- and post-confluent Caco-2 and HT29-MTX mono- and co-cultures were unchanged after 24 h exposure. The impact of increased concentrations of CMOF on cellular metabolic activity remains to be determined. However, at a concentration of 4 mg/mL, the CMOF preparation contained approximately 0.2 and 0.8 mg/mL of neutral and acidic oligosaccharides respectively. A previous study has reported that similar concentrations of purified neutral and acidic oligosaccharide preparations results in proliferation inhibition of Caco-2 and HT29 cells [4]. For example; proliferation of pre-confluent Caco-2 and HT29 cells were reduced after incubation with neutral oligosaccharides (0.2 and 2.3 mg/mL), and also acidic oligosaccharides (0.7 and 0.3 mg/mL) respectively [4,77]. Also, proliferation rates of pre-confluent HT29 cell cultures were reduced after incubation with neutral and acidic oligosaccharides (0.2 and 0.4 mg/mL respectively) from human milk, although Caco-2 brush border-expressing cultures were unaffected at these concentrations [77]. However, proliferation of primary human foetal intestinal cells when incubated (72 h) with 1 mg/mL lactose and tri-galactose was unchanged [78]. In contrast, proliferation of the same cell line, was increased after 48 h exposure to whey produced from bovine colostrum at concentrations ranging from 0.3% to 10% *v*/*v* [79] and also complete human milk at concentrations ranging from 1% to 10% *v*/*v* after 24 h exposure [80]. Thus, it could be hypothesised that neutral or acidic oligosaccharides, as purified preparations, are able to elicit a greater response in inhibiting cellular proliferation than samples comprised of combinations of acidic and neutral oligosaccharides in conjunction with other saccharides components. Whether this could also be related to their mechanism of action requires further study; previous reports indicate that neutral and acidic oligosaccharides induce growth inhibition in intestinal epithelial cell cultures through changes in EGFR signalling and gene expression of cell cycle regulators [59,78]. In contrast, complete human milk increases cell proliferation but not through the EGFR signalling but via a unique tyrosine kinase pathway [80].

## 5. Conclusions 

We have shown that CMOF can improve barrier function of epithelial cell co-cultures that have similar cell compositions to that of the human small (90:10) and large (75:25) intestine *in vivo*. CMOF, at the maximum concentration (4.0 mg/mL), enhanced intestinal epithelial barrier integrity by increasing TEER, mucin gene expression and mucin protein abundance, all of which are essential components of host mucosal function. Thus, CMOF may have important implications as a potential functional food for improving intestinal health.

## Figures and Tables

**Table 1:** The carbohydrate composition, as a percentage of total carbohydrates, of the caprine milk oligosaccharide-enriched fraction (CMOF) obtained after purification. Oligosaccharide content was determined by LC/MS analysis while glucose and galactose content was determined from HPLC analysis.

Carbohydrate	Percentage (%)
Galacto-oligosaccharide	0.4
Lactose	46.1
Glucose	12.0
Galactose	15.9
Oligosaccharides	25.6

**Table 2:** Abundance of oligosaccharides as a percentage of total oligosaccharides present in the CMOF.

Oligosaccharide Common Name	Type	Percentage (%)
α-3′-Galactosyl-lactose or β-6′-Galactosyl-lactose	Neutral	15
6′-*N*-Acetylglucosaminyl-lactose	Neutral	3
6′-Sialyl-lactose or 3′-Sialyl-lactose	Acidic	22
6′-Glycolyl-neuraminyl-lactose	Acidic	42
Disialyl-*N*-lactose	Acidic	18
